# Georgia State Dental Society

**Published:** 1890-01

**Authors:** 


ARTICLE II.

### GEORGIA STATE DENTAL SOCIETY.

TWENTY-FIRST ANNUAL MEETING.

[Reported by "Mrs. M. W. J."]

Continued from page 350, December Number, '89.

Thursday, June 13. Called to order by the President. Dr. D. D. Atkinson, Secretary *pro tem.*

Dr. Browne's resolution came up again, and, after a brief discussion, Dr. Gale, the dissenting member, withdrew all objection.

Dr. Holland moved, as an amendment, that the Examining Board pay the initiation fee of those who have just paid their $10.00 fee.

Dr. Thompson moved as a substitute that the Society accept due bills for the initiation fee.

Dr. Thompson's substitute passed unanimously.

Reports of standing committees:

ANATOMY AND SURGERY.

Dr. R. B. Adair, chairman of the committee, read a paper entitled " Alveolar Abscess Treated Surgically."

At the conclusion of the reading, Dr. Adair exhibited a number of cases of Orthodotia and the apparatus used in regulating.

DISCUSSION.

Dr. Holland—What did you say the deposit on the apex of the root was?

Dr. Adair—I did not undertake to say.

Dr. Holland—I think it is a deposit from the blood—a sanguinary deposit. Dr. Holland fills roots with gold rolled very thin on a broach, scarcely increasing the size of the broach. With soft material you don't know where you are. In one case, when the root was apparently perfectly filled, it abscessed a year afterward, and was treated in vain for three years. After extraction sanguinary deposits were found on the root. After removing the deposits the tooth was replanted, and though the patient removed the splints too soon, it seems to be doing well.

Dr. Coyle—Treatment ofter fails because of necrosis without sanguinary deposits. Extraction and removal of necrosed portion and replanting is the only cure.

Dr. Holland—Does Dr. Coyle mean that when the nerve is dead the tooth is not dead unless necrosed?

(Dr. Coyle apparently did not hear this question, and made no reply.)

Dr. E. T. Adair—In a case where there was an apparent outgrowth from a left superior canine, the gum was dissected away and the growth found to be a perfectly formed tooth, with a second similar but smaller growth.

(Dr. Holland here corrected himself, substituting sanguineous for sanguinary.)

Dr. White (Dr. Carpenter in the chair,) asked if Dr. Adair treated his cases daily.

Dr. Adair replied that he did; otherwise the fistulas would close; syringes with peroxide of hydrogen.

Dr. White, referring to Dr. Adair's use of spoon-shaped instruments for the removal of necrosed bone, asked him if he did not use burrs.

Dr. Adair—Yes; there is nothing better. In the case described he was certain that a portion of the root had been absorbed; he therefore opened the gum sufficiently to smooth and clean off the root, removing all pus, etc., from the cavity. A surgical operation is the only thing that will reach it.

Dr. Carpenter—In treating from the outside, was the root canal filled?

Dr. Adair—In the case described, it was not filled permanently until after the surgical operation.

Dr. White—In ninety-nine cases out of a hundred one thorough injection, using creosote and iodine, will be sufficient; then fill the tooth, and in a week there will be no trace of the fistula left. It can all be done at one opening if there is a fistulous opening. We may feel perfectly safe if we can pump our medicine through. Even without pumping through, if we can fill perfectly to the apex, nature will heal the fistula.

Dr. Browne—Remove the cause and a cure will follow.

Dr. White—For a necrosed tooth or a necrosed bone, nothing equals a sharp burr in the engine. You can at once tell when you strike sound bone. For regulating, Dr. White uses the same kind of plate for every case—a vulcanite plate having a band on the outside covering the incisors, bicuspids and molars; also a band on the palatine surface. In reply to a question from Dr. Carpenter, Dr. White stated that he never allows a metal plate to touch the natural teeth. In the case of a child having a sore nose, the right lateral and left central incisor were touching, with no room for the right central, which nowhere could be detected. There was, however, a painful protuberance in the nostril. The incisors were separated by means of a suitable plate. After two weeks the soreness had much diminished, and ten days later a well defined cusp was perceptible in the vacant space, but very high up; the missing right central came down into its place all right. The crowns of the posterior teeth can be covered with the plate to open the bite when necessary, pieces of rubber placed over the tooth under the plate, on either of the palatine or buccal surface, etc. The rubber blocks will usually give sufficient pressure without ligatures, which are liable to injure the gum.

Dr. Robinson—How do you distinguish between true and dead bone?

Dr. White—It is similar to cutting cheese and coming to the rind.

Dr. Adair had hoped to hear more discussion of the treatment of chronic abcess and oral surgery. In chronic cases with necrosed bone, you cannot cure by pumping through once and filling the canal; only the simplest cases yield to that treatment. If the root is absorbed and the alveolus necrosed, a surgical operation becomes necessary. The subject of *immediate root filling* has much been discussed. The idea was at first hooted and scoffed at, but the practice is now almost universal, and is considered good, sound practice. When a pulp is devitalized and removed, the canal should be dried and filled at once. Two years ago Norman W. Kingsly thought nothing could equal the jackscrew for moving or rotating teeth, but he now uses ligatures, spiral springs, etc. A valuable appliance for regulating consists of a platinum band to which are soldered little cylinders in which are slipped the ends of a coil of spring wire. Almost any tooth can be rotated in two or three days with very little soreness or annoyance.

Dr Carpenter—Some one said it was impossible to cure chronic abscess of the six interior teeth as to fly. Dislikes to hear any one use the word impossible—the most should be, " in my hands it is not a success." Twenty years ago he had a case in which the nerves of the four front teeth were dead and gone, with fistulous openings so that when you syringed through any one it would pass out through the other three, and had been so for ten years. The face had swollen and gone down again over and over again. After pumping pure wood creosote through and through, the four teeth were filled that day. The patient has been seen often during the twenty years that have passed, and there has never been any return of the discharge. In my hands—with the help of nature and Providence—can cure chronic abscess of the six anterior teeth, except where there are constitutional or hereditary complications. After the first treatment fill the cavity and the abscess will cure itself.

Dr. Holland said he had a similar case of eight years' standing. He had filled the teeth at the first sitting, and had no further trouble.

Dr. McElhaney—The question has been asked: What constitutes a chronic case?

Dr. Adair—Where the alveolar tissues are all destroyed, the apex destroyed, with sharp, jagged points. I have treated such cases for weeks and weeks, and they have been treated for years without healing.

Dr. Cagle—If you had done at first what you did at last, would you not have succeeded earlier?

Dr. Adair—Of course. When I did the right thing, I succeed, and that was the surgical operation, but not the simple treatment that has been described by others.

Dr. Boozer—Diagnosis of the pus will show the cause of the trouble and indicate the treatment. One treatment will not cure in certain phases, or with a certain character of pus which flows where nature cannot throw off the diseased tissues.

Dr. Colding—I must be behind the times, for I prefer the jackscrews to ligatures in regulating in many cases. There are so many failures in implantation among the great operators in large cities that we lesser lights in the smaller towns had better not undertake it.

Dr. Brown—I wish to protest against the huge rubber plates often used in regulating. Simple bands with cylinders soldered to them, and ligatures properly applied, cause no discomfort to the patient.

Dr. Holland—A wedge between the tooth and the plate carries it just so far and then allows it to rest, and does better work than piano wire springs and jackscrews, which occasion a continual strain. The wedge gives nature time to build up and recruit, with less danger of inflammation.

Dr. White—A method, or an appliance, should not be condemned unless we know it is not good in any case.

Dr. D. D. Atkinson—After you get the teeth into place, what then?

Dr. Adair—Use a retainer and hold them in place until they are made firm and strong by new bone deposits.

OPERATIVE DENTISTRY.

There being no report from this committee, and no paper, Dr. D D. Atkinson asked for a discussion of the subject of

COPPER AMALGAM,

Based on the following verbal report of a case in practice:

An ulcer on the upper lip, with a discolored tooth in contact underneath. The ulcer was touched with nitrate of silver. After two weeks the tooth was found to be quite green. The patient said that the tooth had a screw post in it, apparently of copper wire.

Dr. Bouton thought it might have been a Howe screw-post. The tooth was perfectly translucent—as clear as a piece of glass, and perfectly preserved. The filling was removed and temporarily filled with gutta-percha. The filling was a very handsome gold filling, with a screw-post perfectly imbedded in it; the apex was filled, probably with cement, and was not removed, because it was doing well. Dr. Atkinson said he was waiting to learn how to restore the tooth to its color. The ulcer had healed, but had left a blue-black cicatrix. What force sent this clear green-glass color all through the tooth?

Dr. Wardlaw said he had had a similar case apparently, also caused by a screw-post. In less than a week the patient had a green tooth. He now has another similar case. In the March *Cosmos* Dr. Butler says it occurs from the use of white metal or pure nickel.

Dr. Hopps—The Howe posts in contact with dentine, in some patients, gives this green color; does not think it can be bleached.

Dr. Atkinson—In the case described the ulcer fitted the tooth like a hull to a nut; it looked as though some exudation from the tooth had caused the ulcer. He wished for information as to the cause of the discoloration and the means of bleaching the tooth.

Dr. Boozer thought there must have been moisture between the post and the tooth.

Dr. McElhaney said this discoloration was not uncommon where screw-posts were used, but if the tooth was doing good service he would not disturb the filling.

Dr. Adair—We have had no evidence in the case cited that the tooth was not discolored before the post was inserted.

Dr. Atkinson—I learned that the tooth was dead, but good in color, when the post was inserted.

Dr. Adair had seen the same color in teeth which had no post. In one case he had filled a tooth with gutta-percha and chloro-percha, and the crown with gold In two years it took on exactly the appearance described by Dr. Atkinson. He took the filling out, and tried every method known to him to bleach it, but with only partial restoration, the tooth going right back to the same green color. He then lined the cavity next to the labial wall with chalk moistened with alcohol, and filled with cement, finishing with gold. This improved its appearance somewhat, though it was not entirely restored in color. Bleaching is a humbug, and we should be very careful in the use of chemicals in a tooth.

Dr. Roach had found that the same posts used with amalgam caused no discoloration; thought it must be the result of some chemical action between gold and the post.

Dr. Gayle thought the facts seemed to suggest a vague theory of galvanized action.

On motion, the subject was passed.

MECHANICAL DENTISTRY.

No paper.

Dr. Thompson hoped the subject would not be passed without discussion. He thought the Ward electro-metallic plates destined to work a revolution in this department. The only condemnation comes from Dr. Haskell, who probably has not given it a trial. They certainly fit the model more perfectly than swaged plates can do, except in such exceptionally skilled hands as those of Dr. Haskell.

Dr. Holland—Rubber is to the poor man what the hickory shirt is ; for the rich man, of course, nothing equals gum. Give it to all who can pay for it, and show them in what it is the best. If we would invariably do this we would raise prosthetic dentistry to a higher plane.

Dr. Adair—Shall we let this important practical subject pass without any remarks ? Are we getting too big, too important, too scientific to discuss practical matters ? I am trying to teach my patients the difference between a $50.00 and a $10.00 set of teeth ; or as Dr. Holland puts it, between the fine linen and the hickory shirt. Try convincing them that there is something better than rubber. We are, or should be, the educators of the people. If we tried, all could get plenty of metal work. I have quite recently made seven gold plates, and think a gold plate with rubber attachments equal to continuous gum. With plain teeth and pink rubber you can do as artistic work as with continuous gum, restoring the contour of the face quite as perfectly. The work is much lighter, thinner in the roof of the mouth, and does not interfere with articulation. Continuous gum is too liable to accidents, and the least crack admits the oral secretions. As to the bridge business, we had better go slow till the International Tooth Company is settled.

Dr. Holland—I cannot agree that gold plate with pink rubber attachments, is in all respects equal to continuous gum. Certainly not in cleanliness. Take a tooth off any rubber plate and hold it in the flame and note the effluvia! There is the weak point. Pink rubber will not long retain its perfect colors—another weak point. Continuous gum is all the same material as the tooth itself, and unites perfectly with it. It is absolutely the most cleanly work possible, and also the most comfortable. A gold plate is a little lighter, but this is not perceptible when in the mouth. If the fit is good the patient will never complain of the weight. Full plates of Weston's metal are very heavy, but this is no cause of complaint from the wearer.

Dr. Thompson was surprised that celluloid has not been mentioned. It has had a hard fight. Many took it up merely to dodge the rubber license—the Goodyear patients—and did poor, cheap work with it, bringing it into unjust disrepute. It is far superior to pink rubber, and in artistict possibilities fully equals continuous gum. To get its best results it should be pressed in dry heat, between metal surfaces. It makes beautiful artistic work, light in weight, perfect in color, capable of most artistic possibilities in restoration of contour, etc.

The mucous membrane never becomes inflamed or honey-combed, as under rubber. It has no offensive smell; you all know the vile odor of an old rubber plate. It will last quite as well as rubber, and keeps sweet and clean to the last. When it gives way, it gives way all over, but is easily duplicated.

Dr. Holland said that he appreciated celluloid sufficiently to wear it himself. It is true that it crawls—out of his mouth and on to the pillow at night, and he never knows how it gets there—but any other material might do the same thing.

Dr. Gayle says that in his experience, it turns black, and looks as if burned.

Dr. Thompson thought it was probably burned before the patient got it! If cooled under pressure it will never change shape.

Dr. Carpenter said he had used rubber from its first introduction, and considers it a great blessing to the people, though a poor material in itself, compared with others. It undoubtedly produces catarrhal, bronchial and throat affections, and is injurious to the mucous membrane. Believes celluloid is one hundred per cent. better than rubber. There is nothing injurious in it, and he removes rubber plates, replacing them with celluloid to great advantage.

Dr. Thompson said he had undoubtedly cured incipient catarrh, sore throat, and bronchial affections by removing rubber plates and substituting celluloid. In one case the patient had spent hundreds of dollars in vain seeking relief from throat trouble and indigestion. In having new teeth filled he incidentally mentioned the other troubles. A rubber plate of three teeth was replaced by a piece of bridgework, and the other troubles entirely disappeared in less than three weeks. He was the happiest man alive, and has never had any return.

Dr. White said that for regulating, instead of a cumbrous plate covering the entire roof of the mouth, he uses gold wire, quarter of an inch wide, making a skeleton plate, which is very comfortable and very strong.

Dr. Holland : A spring plate ?

Dr. White : No, a perfect fit, but not coming in contact with the natural teeth, which, in a certain character of teeth, is injurious to them.

Dr. W——— described a crown placed on a bicuspid root, broken off on a slant above the anterior process. An impression was taken in premium gutta-perch. The two canals of the root were utilized for anchorage. A canine tooth was used, which was built out to the form of a bicuspid. The patient had worn a small plate which irritated the free margin of the gum. This was relieved by setting the crown well up under the gum.

Dr. B——— had had a similar case, where a pivot tooth had split the root, splitting off the outer portion under the process. . A gold band was fitted to the root with a lip covering the broken portion ; the edge of the lip was bent back and a pin passed through it. The crown was held with cement.

Dr. Adair: How did you prevent hemorrhage?

Dr. Browne: Waited till it stopped; then washed it out and dried, when it was ready for the cement.

When a root, covered up in the gum, is found to be good and solid, the soft tissues can be pushed back with cotton or gutta-percha, to give access, and a pin inserted, building around and down with amalgam sufficiently to receive a crown, which can be made to give good satisfaction. One patient is wearing seven such crowns, which are giving good satisfaction.

Dr. White said that much of the fine work described in the journals require too much time and labor, especially for a man who employs no assistant, using only his own two hands.

Dr. Browne thought no better work could be found for our hands to do.

Dr. White said it would necessitate disappointing too many other patients.

Dr. W——— described a case in which an inferior under tooth and first bicuspid were capped and a block of gold, slightly curved, swung in to fill the place of the missing tooth, articulating well and answering all the purposes of mastication, a heavy mustache and whiskers preventing any exposure of the gold.

Dr. T——— described a similar all metal bridge from the superior cuspid to a dead third molar. Patrick's seamless crowns were stamped and filled with twenty karat solder and all solder together, making a very satisfactory piece of work.

Dr. W——— uses the platinum pins from broken teeth in filling such crowns, saving solder.

Dr. A——— asked if this work was made to rest on the gums.

Dr. ——— It is not allowed to touch the gums; self cleansing space is left. There is no porcelain in this kind of work to break or give way. We don't realize what we can do with old roots until we try. It may require several days to compress and pack to get the gums away ; the floor of the cavity may be broken down between the roots so that it has to be restored with gold, but even with such roots may be restored and give great satisfaction to a patient who does not want a plate or a bridge. The Logan crown, used with a ferule, can be fitted accurately to any root, trimming it down to the gum on the anterior margin; leaving the ferule on the root, fit the crown into it and fasten it with cement. The root must be so shaped that the ferule will fit accurately, scaling off the enamel or shaping the cementus so that there will be no space between the ferule and the root towards the apex. If the root is broken off high up, it can be filled and built down with amalgam, and the ferule fitted up to the amalgam stump. It makes water-tight work like a coffee-dam, strengthens the root and prevents decay. Even when the roots are entirely separate, they can be banded together and make a good foundation for a gold crown.

Dr. McElhaney presented the claims of the Dental Protective Association.

On motion, subject passed. Adjourned to 3 P. M.

---

Thursday, 3 P. M. Called to order, the President in the chair.

Dr. Holland moved a suspension of the rules and the election of officers.

Dr. Catching said that after the election of officers the meeting would virtually be broken up, and moved that the regular order of business be taken up for an hour.

Dr. Whitaker did not want to wait till after every one was gone and only a few left to transact important business.

On motion of Dr. Catching the election of officers was made the special order of business for 5 o'clock.

Report of standing committees:

DENTAL EDUCATION AND LITERATURE.

Dr. Catching, chairman of the committee, made a verbal report on the wonderful improvement recently made, both in standard and journalistic literature, which now compares favorably with the highest standards. He said that there are four means or methods of education. 1st. The standard literature, which is the basis or foundation of all professional attainment, and that by which we are judged. 2d. Journalistic literature, through which all may keep abreast of the times and note the great advances making month by month. 3d, Our colleges—institutions of learning which now show a growing tendency to higher standards. Boards of education are taking a more advanced stand. 4th. Association work. This should be considered our highest privilege, but it makes the heart sad to see the lack of interest manifested by those most in need of the elevating influences of society meetings. No man can keep abreast who ignores this source of improvement.

Dr. H. S. Colding, of the committee, read a paper entitled

DENTAL EDUCATION.

Dr. Catching made a supplemental report, in which he spoke of his pride in the realization of his prophecy of a post-graduate school. Also, the necessity to the dentist of purchasing books and having at hand the latest additions to our professional literature. He said that it was to be regretted that a great house, which year after year makes a fine exhibit of dental specialties, never hearing of the recently published works of so much importance to our profession.

On motion, the subject was passed.

### VOLUNTARY ESSAY.

Dr. Catching read a paper entitled, "SOME THINGS I DO; SOME THINGS I DON'T DO."

Dr. W. G. Browne read a short paper, entitled "A FEW POINTS CONFIRMED BY EXPERIENCE."

Discussion.

Dr. R. B. Adair thought that instead of building up two approximal surfaces in one solid mass of amalgam and then filing across, it would be better to take a little more trouble and make two buildings knuckling as nicely as in gold.

Dr. Winkler had found, in the treatment of bad teeth, that if bichloride of mercury goes through the apex, it gives severe toothache. He was inclined to distrust copper amalgam, not knowing much of the nature of the salts of copper and what their effects might possibly be.

On motion, subject passed.

### REPORT OF SPECIAL COMMITTEE ON ADDRESS TO THE PUBLIC.

Dr. Wardlaw, the only member of the committee present, said that not having been able to obtain any information as to what was wanted or intended, he had done nothing in the matter.

President White said that the President's address of last year bore so strongly upon that subject that the committee had been appointed in deference to his views, and he regretted that nothing more had been heard from him on the subject.

Dr. Catching asked for an expression of views on the matter of the exclusion of the members of the Examining Board from participation in the proceedings of the society.

The Board included some of the best members of the society, and they were greatly missed.

Dr. Colding thought that they might arrange it so that one could stay with the applicants to improvise their work, attending the general meeting in alternation.

Dr. White said it had been suggested that the Board meet all day Monday, getting through on Tuesday during the transaction of preliminary work.

Dr. Carpenter said that the Executive Committee had not informed them if arrangements had been made for meeting on Monday.

Dr. Coyle—State societies all over the country are appointing delegates to attend the International Dental Congress in Paris. He thought the Georgia State Society should fall into line.

The President said that any members intending to attend the Congress would receive certificates as delegates.

Dr. Catching hoped the society would not endorse the Congress, which is a private enterprise and a direct thrust at the Dental Section of the International Medical Congress. The dental societies of other foreign nations are taking no part in it, and he hoped the Georgia Society would not endorse it officially.

Dr. Coyle—No man refuses to go to a feast because it is a private party, if he is assured he will have a good time. He failed to see the force of the objections raised.

The Committee on New Appliances reported a very fine exhibit from the S. S. White Manufacturing Co., in charge of Mr. Selby; Dr. W. G. Browne, rubber disks for the protection of paper disks, etc.; also, his universal rubber-dam clamp with adjustable jams tightened with a screw; Dr. B. S. Burns, engine mallet, which was used by Browne in his clinic with the greatest satisfaction to both operator and patient.

On motion, the sum of $30.00 was ordered paid " Mrs. M. W. J." as reporter of proceedings; also, as her expenses had been unusually heavy, that the Transactions be donated to her, to be disposed of by her to the best advantage for her own profit, with the suggestion that if given to the *Southern Dental Journal*, the official organ of the Society, she would receive $25.00 additional for the same.

Dr. R. B. Adair hoped some change would be made in the time of meeting of the Examining Board. As it now stands, five of the best men of the Society are worked to death and prevented from attending the meeting. He would suggest that they have a special meeting in May for the purpose of examining applicants.

Dr. McElhaney stated that a resolution had been passed at the Macon meeting that the Board should meet two days earlier than the Society.

Dr. Adair—They tried that once, but no students appeared. If a meeting is held for that special purpose, they would be obliged to appear before the Board.

Dr. Carpenter—No motion is necessary. They can meet whenever they choose.

Five o'clock, the hour set apart for the election of officers having arrived, Dr. R. B. Adair, of Gainesville, was nominated for President by Dr. Holland ; seconded by Dr. Wardlaw.

Dr. S. B. Barfield was nominated by Dr. D. D. Atkinson ; seconded by Dr. McElhaney.

The election resulted as follows :

President—Dr. S. B. Barfield, Macon.

First Vice-President—Dr. R. B. Adair, Gainesville.

Second Vice-President—Dr. W. G. Browne, Atlanta.

Recording Secretary—Dr. C. A. Ryder, Gainesville.

Corresponding Secretary—Dr. L. D. Carpenter, Atlanta.

Treasurer—Dr. H. A. Lowrance, Athens.

Examining Board—Drs. J. H. Coyle, G. W. McElhaney, W. C. Wardlaw, A. G. Bouton, G. W. H. Whitaker.

Executive Committee—Drs. N. A. Williams, S. B. Adair, H. S. Colding, G. G. Holland, W. W. Hill.

Dr. R. B. Adair nominated for the place of meeting, Gainesville, and presented very tempting inducements—special invitation from the Mayor, another from the most prominent ladies of Gainesville, a grand banquet tendered by a prominent citizen, an excursion to Tallulah Falls, for which special terms were offered by the railroad, etc.

Dr. McElhaney called attention to the fact that by law the meeting must be held in Atlanta, unless changed by a two-thirds vote.

Gainesville was unanimously voted for the place of next meeting, to be held the Second Wednesday in July, '90.

The following delegates were appointed:

To the American Dental Association—Drs. H. T. Colding, G. H. Winkler, J. D. Lanier, A. G. Bouton, L. D. Carpenter.

To the international Dental Congress—Drs. J. H. Coyle, G. W. McElhaney, S. A. White, A. G. Bouton, W. C. Wardlaw.

After the usual ceremonies of installation of officers elect, adjourned to meet in Gainesville, July, 1890.—*Southern Dental Journal.*

